# BOLD Granger Causality Reflects Vascular Anatomy

**DOI:** 10.1371/journal.pone.0084279

**Published:** 2013-12-13

**Authors:** J. Taylor Webb, Michael A. Ferguson, Jared A. Nielsen, Jeffrey S. Anderson

**Affiliations:** 1 Department of Bioengineering, University of Utah, Salt Lake City, Utah, United States of America; 2 Program in Neuroscience, University of Utah, Salt Lake City, Utah, United States of America; 3 The Brain Institute, University of Utah, Salt Lake City, Utah, United States of America; 4 Division of Neuroradiology, University of Utah, Salt Lake City, Utah, United States of America; Cuban Neuroscience Center, Cuba

## Abstract

A number of studies have tried to exploit subtle phase differences in BOLD time series to resolve the order of sequential activation of brain regions, or more generally the ability of signal in one region to predict subsequent signal in another region. More recently, such lag-based measures have been applied to investigate directed functional connectivity, although this application has been controversial. We attempted to use large publicly available datasets (FCON 1000, ADHD 200, Human Connectome Project) to determine whether consistent spatial patterns of Granger Causality are observed in typical fMRI data. For BOLD datasets from 1,240 typically developing subjects ages 7–40, we measured Granger causality between time series for every pair of 7,266 spherical ROIs covering the gray matter and 264 seed ROIs at hubs of the brain’s functional network architecture. Granger causality estimates were strongly reproducible for connections in a test and replication sample (n=620 subjects for each group), as well as in data from a single subject scanned repeatedly, both during resting and passive video viewing. The same effect was even stronger in high temporal resolution fMRI data from the Human Connectome Project, and was observed independently in data collected during performance of 7 task paradigms. The spatial distribution of Granger causality reflected vascular anatomy with a progression from Granger causality sources, in Circle of Willis arterial inflow distributions, to sinks, near large venous vascular structures such as dural venous sinuses and at the periphery of the brain. Attempts to resolve BOLD phase differences with Granger causality should consider the possibility of reproducible vascular confounds, a problem that is independent of the known regional variability of the hemodynamic response.

## Introduction

A topic of great interest in recent literature is the creation of a directional connectome based on functional connectivity MRI (fcMRI) data. fcMRI is based on the observation that different brain regions show synchronized blood-oxygen-level-dependent (BOLD) time series that correspond with established functional neuroanatomy[1]. Recent work has established consistent relationships across large numbers of subjects that show a canonical organization of brain network architecture that reflects extensive prior work characterizing regional brain function[2-4].

Yet most of the attempts to characterize a whole-brain connectome have been correlational, modeling mutual relationships between brain regions rather than determining the extent to which the communication is preferentially unidirectional[2,3,5-8]. One technique that has been proposed for determining directional interactions is to measure sequential activation in the brain using functional MRI. For task-based fMRI, a phase difference between two brain regions may suggest, though not prove, a causal relationship between the two regions. In task-free, or undirected cognition, it is possible that similar relationships persist.

Exemplifying this approach are techniques such as Granger causality or vector autoregression, which ascertain whether the future of a time series can be more accurately predicted given past values of another time series than by using the past values of the time series in question alone[9,10]. These techniques have been applied to fMRI data[11] as a method for establishing sequential neural activation associated with directed causality[12]. When examining two time series, A and B, if past timepoints for signal A and B allow better prediction of future timepoints of signal B than by using past timepoints of signal B only, then A is said to have a Granger causal relationship with B[13]. The use of methods allowing inferences of directed neural communication has been the subject of several recent reviews[14-16]. Another method that has been proposed for determining temporal influences of one BOLD time series on another is total interdependence, which considers temporal influences of two time series on each other, as well as co-varying common input[17]. This method has been shown to elucidate resting state networks that more precisely correspond to contemporaneously acquired task activation maps[17].

This set of techniques has even more recently become relevant to connectomics given the advent of large-sample datasets of thousands of subjects[5,18]. Since BOLD time series are noisy, and at best small statistical relationships can only be inferred through large datasets, it is hopeful that the use of such large datasets might provide an opportunity for large-scale analysis of Granger causal differences in resting state data with higher statistical power. Reproducibility of correlative measurements improves with 1/square root of imaging time and/or number of subjects, so reliability of such measurements is likely to be better assessed using available large samples[19]. 

Yet lag-based analysis techniques such as Granger causality have recently become the topic of controversy. Their accuracy in fMRI studies has been questioned in a study using simulated fMRI data to evaluate the accuracy of inferences about directed functional connectivity[20]. Subsequently, it has been suggested that a constraint for many of these investigations could be the relatively small sample size of the data set, and perhaps using this technique on a larger data set could overcome the limitations of poor temporal resolution[21], particularly since lag-based simulations were more accurate for longer sessions (60 minutes) compared to shorter sessions (2.5 minutes)[20]. Other studies have examined the effects of temporal sampling on Granger causality inferences and found that poor temporal resolution of acquisition and measurement noise may lead to incorrect inferences, although Granger causal inferences appear to be robust to variations in hemodynamic response properties[22]. We attempted to test whether a large sample of resting state fMRI data might be able to predict directed functional connectivity relationships by examining a high resolution spatial matrix to determine whether the spatial relationships conform to known directional connections in the brain, taking advantage of the statistical power afforded by large datasets. 

## Materials and Methods

### Ethics Statement

All analyses and data collected for this study were performed in accordance with guidelines established by the University of Utah Institutional Review Board. Data from anonymized publicly available datasets were all shared in accordance with guidelines established by human subject protection boards of the the corresponding institutions as described on the project websites.

### fMRI Data Sources

1240 subjects were analyzed from publicly available datasets released with the open-access 1000 Functional Connectomes Project (http://fcon_1000.projects.nitrc.org/, FCON1000) in which resting-state fMRI scans have been aggregated from 28 sites [5] as well as typically developing subjects from the ADHD 200 project from the International Neuroimaging Data-sharing Initiative (http://fcon_1000.projects.nitrc.org/indi/adhd200/) including 8 sites[23]. For inclusion we required that subjects’ ages were between 7 and 40, with BOLD whole-brain coverage from MNI coordinates z=-35 to z=70. Any subject for whom postprocessed data did not cover all 7266 ROIs used for this analysis was discarded prior to analysis. For inclusion, all subjects included an MPRAGE anatomic sequence that was successfully segmented and normalized to MNI space. Although postprocessing steps were performed using an automated batch script, the results of normalization, segmentation, and realignment steps were manually inspected for all subjects, and any subject for whom the normalized and segmented images were not in close alignment with the MNI template on visual inspection were discarded. The datasets from which subjects met all criteria are listed in Table **1**. The mean age of all subjects was 19.2 +/- 6.6 s.d. years (range 7-39). 695 subjects were male; 545 were female. The subjects were divided into two groups of 620 subjects that did not differ in age (19.1 +/- 6.6 s.d. years and 19.2 +/- 6.6 years, p=0.96, two-tailed t-test) or gender (p=0.68, two-tailed t-test) and analyses were performed separately for the two groups as a replication sample as well as for the full set of 1240 subjects. All subjects' data were processed in the same manner regardless of the site from which they were obtained.

**Table 1:** Sources of 1240 open access resting state datasets used for analysis.

**Site(FCON 1000)**	**n**	**TR (s)**	**# of Volumes**	**Site(ADD 200)**	**n**	**TR (s)**	**# of Volumes**
Ann Arbor	18	1	295	Kennedy Krieger	61	2.5	124
Baltimore	22	2.5	123	NeuroImage	21	1.96	261
Bangor	1	2	265	NYU	89	2	176 - 352
Beijing	188	2	225	OHSU	40	2.5	234
Berlin	25	2.3	195	Peking	112	2	236
Cambridge	193	3	119	Pittsburgh	82	2	196
Cleveland	12	2.8	127	Washington U	50	2.5	133 - 396
ICBM	17	2	128				
Leiden	31	2.2	215				
Leipzig	36	2.3	195				
New York	47	2	192				
Newark	17	2	135				
Orangeburg	7	2	165				
Oulu	100	1.8	245				
Oxford	14	2	175				
Palo Alto	10	2	235				
Queensland	17	2.1	190				
Saint Louis	30	2.5	127				

An additional dataset released by the Human Connectome Project (HCP)[24,25] was tested consisting of 80 subjects for which both resting state (4 sequences of 1200 volumes each per subject, TR=720 ms, multiband BOLD acquisition[26,27], 2x2x2 mm spatial resolution) and task paradigm acquisitions were available. Data were provided by the Human Connectome Project, WU-Minn Consortium (Principal Investigators: David Van Essen and Kamil Ugurbil; 1U54MH091657) funded by the 16 NIH Institutes and Centers that support the NIH Blueprint for Neuroscience Research; and by the McDonnell Center for Systems Neuroscience at Washington UniversityTask paradigms are as described on the human connectome project website (www.humanconnectome.org) and included an emotional, gambling, language, motor, relational, social, and working memory task with the same acquisition parameters as the resting state data. Each task was performed twice for each subject with left-right and right-left phase encoding directions.

### fMRI Preprocessing

The following sequence was used for image preprocessing of FCON 1000, ADHD200, and single subject BOLD image datasets. Using SPM8 toolbox (Wellcome Trust, London), BOLD images were realigned (realign, estimate and write), slice timing corrected, coregistered to MPRAGE image (coregister, estimate and write), and normalized to MNI template (normalize, estimate and write, T1.nii template). Gray matter, white matter and CSF were segmented from MPRAGE image using SPM8 segment function (modulated, normalized, thorough clean). Images were bandpass filtered between 0.001 and 0.1 Hz and a linear detrend was performed at each voxel in the brain. No additional temporal smoothing was performed beyond that entailed within the initial slice timing correction. Time series were averaged from 2 ROIs in the white matter (bilateral centrum semiovale, CSF (lateral ventricles), soft tissues of the head and face, and 6 rigid motion correction parameters from realignment step as previously described[28] and for each voxel, a general linear model was used to find a best fit for white matter, CSF, soft tissues, and motion parameter time series, which were subtracted from the voxel’s time series. No regression was performed of the global signal or gray matter[28-31]. No spatial smoothing of the data was performed throughout preprocessing. Removal of frames with residual motion[32] was not performed for this analysis because this would result in frameshift errors in calculating lag-based cross-correlograms of time series between subjects. 

For Human Connectome Project task data, a preprocessing pipeline was used that consisted of a minimal preprocessing pipeline[31] implemented in the FSL software library[33]. For HCP resting state data, an extended preprocessing pipeline was used that included the ICA-FIX de-noising procedure (“Resting State fMRI FIX-Denoised Extended Release”)[34]. This technique includes an automated independent component analysis decomposition of each subject’s fMRI data, with removal of “bad” noise components from the data resulting in cleaned images.

### Regions of Interest Used

In order to evaluate the spatial distribution of Granger causality differences in the gray matter, we used a set of ROIs that covered the gray matter at 5 mm resolution. A gray matter restriction mask was obtained by selecting voxels at 3x3x3 mm resolution where the SPM8 grey.nii image showed intensity > 0.3. Beginning with the right, inferior, posterior corner of the image, voxels were retained in the image if they were greater than or equal to 5 mm Euclidean distance from previously retained voxels. This yielded 7266 seed voxels, each separated by at least 5 mm. The gray matter restriction mask was then parcellated into 7266 ROIs, based on which of the seed voxels was closest to any given voxel in the restriction mask[35,36]. The ROIs ranged from 2 to 12 voxels in extent (mean 4.9 +/- 1.3 voxels at isotropic 3 mm resolution). ROI’s had no voxels in common. Because many of the publicly available datasets used in the analysis did not include BOLD data at the vertex or inferior cerebellum, brain voxels with MNI coordinates z<-35 or z>70 were not included in the 7266 ROIs. Centroid locations of each of the ROIs have been previously described[36].

A subset of 264 of these 7266 ROIs containing the MNI coordinates of functional brain network nodes was used as a set of seed regions. These coordinates were derived from a large meta-analysis of functional connectivity data and include hubs of distributed brain networks relevant to the network architecture of the brain[3]. These two sets of ROIs defined a 7266 x 264 matrix of “connections” between each point in the gray matter and key functional regions of the brain.

### Granger Causality Estimates

For each subject, preprocessed BOLD data was used to extract time series for each of the 7266 ROIs, of which 264 seed region time series were a subset. Then for each connection of one of the seed regions to one of the 7266 target regions, Granger causality was measured in both directions to obtain a metric of the extent to which the seed ROI Granger causes the target ROI and the extent to which the target ROI Granger causes the seed ROI. Granger causality estimates were performed in Matlab (Natick, MA) using the Granger Causal Connectivity Toolbox[37], using the *cca_granger_regress.m* function and a model order of 3 lags. A model order of 3 was chosen based on 500 randomly selected pairs of time series, for which *cca_find_model_order.m* was calculated using a Bayesian Information Criterion. The optimal model order results had a mode of 1, with a few connections showing 2 or 3 as an optimal model order, followed by some connections indicating an optimal model order of greater than 20 lags, which was thought biologically implausible. 

To estimate significance of Granger causality estimates, we calculated separately for the entire dataset (n=1240) as well as for each subset (n=620) of subjects a two-tailed nonparametric sign test as to whether the seed ROI Granger caused the target ROI more than the target ROI Granger caused the seed ROI. For the set of 7266 x 264 connections, those connections were significant exhibiting acceptable false discovery rate q<0.05 over all connections.

A similar procedure was performed on the HCP dataset, with calculation of Granger causality estimates for a 7266 x 264 matrix of ROIs for each of the 4 fifteen minute resting state sequences and each of the two task-based sequences for each of 7 tasks for each subject. The Granger causality estimates were averaged for all sequences for the same subject (average of 4 acquisitions for resting state data and 2 acquisitions for each task), and statistical significance was assessed across subjects using nonparametric sign tests resulting in a 7266 x 264 matrix of Z-scores for resting state condition and for each of 7 tasks. Prior to preprocessing, the first 10 volumes of all HCP acquisitions were discarded to prevent artifacts from stabilization of the BOLD signal.

### Regression of Age and Head Motion

Recent work has indicated that even small head movements can significantly affect functional connectivity parameters[32,38]. Although it is not intuitive how head motion would selectively phase advance one ROI compared to another in our data, we performed an additional step to minimize the chance of such influence. For each “connection” consisting of a pair of ROIs, we calculated correlation between the difference in Granger causality between seed and target ROIs and age and motion covariates across subjects. We included subject age, mean head motion (the sum of 6 realignment parameters calculated in the realign step, averaged across volumes), and maximal head motion (maximal displacement of any volume during realignment) as covariates[32]. None of these measurements showed significant covariation using Spearman correlation coefficients with Granger causality differences across subjects after false discovery rate correction.

### Calculation of Arterial and Venous Density Maps

We hypothesized that differences in Granger causality might be influenced by the distribution of vascular structures in the brain. To assess for this possibility, we obtained a retrospective sample of 33 magnetic resonance arteriograms (MRA) and 34 magnetic resonance venograms (MRV) obtained at the University of Utah between 2009 and 2011 which were read as showing no pathology by a board-certified, certificate of added qualification (CAQ) neuroradiologist. The average age of subjects from whom arteriographic and venographic images were analyzed were older than for the fMRI sample (MRA: mean age 53.8 +/- 16.1 years s.d.; MRV: mean age (43.1 +/- 16.4 years s.d.)

Both MRA and MRV images were obtained using a standard clinical time-of-flight angiographic sequences (Siemens, Erlangen) without the use of gadolinium contrast. MRA images were acquired with a 768 x 696 matrix size and 0.5 mm slice thickness in the axial plane and MR images were acquired with 512 x 416 matrix size and 1.5 mm slice thickness in coronal plane. TE, TR, and flip angle varied between scans, which were performed on a combination of 1.5 Tesla and 3 Tesla Siemens scanners. MRV images included the entire brain, and MRA images included only the cerebellum through the cingulate gyrus, designed to image arterial inflow through the pericallosal arteries. For each subject, MRA or MRV source images were normalized to the MNI template brain (T1.nii in SPM8), with resampling of the normalized images to 3x3x3 mm spatial resolution, and manual inspection for appropriate coregistration. Mean signal was normalized for each subject by dividing each voxel's value by the standard deviation of signal intensity across in-brain voxels. These images were averaged to obtain a mean venous and mean arterial density image which was thresholded at 2 standard deviations of signal for in-brain voxels to obtain masks of high arterial and venous density across a population. It is noted that the MRA and MRV images used for analysis are effective only at showing relatively large arteries, veins, and venous sinuses, and do not show vessels of smaller diameter throughout the brain parenchyma.

### Evaluation of Stationarity of BOLD Time Series

To assess the effects of nonstationarities present in the BOLD time series data, we employed a Kwiatkowski-Phillips-Schmidt-Schin (KPSS) test[39] and augmented Dickey Fuller test[40] for stationarity on each subject’s preprocessed BOLD time series in each of the 264 seed ROIs. Analysis was performed using the cca_kpss.m function of the Granger Causal Connectivity Toolbox[37], with a p=0.05 threshold for rejecting the null hypothesis of stationarity (KPSS test) or unit root (Dickey-Fuller test). Although most data passed the KPSS test, a majority of the data did not pass the more stringent Dickey-Fuller test requiring rejection of a null hypothesis of unit root. To more completely evaluate whether potential nonstationarities contributed to the results, the entire dataset was reanalyzed using the difference time series, for which a large proportion of the dataset was stationary by both KPSS and Dickey-Fuller tests. Data subsamples that passed all stationarity tests (41.6% of difference time series) were compared to those that failed one or more tests (58.4%, almost always the Dickey-Fuller test). Across the dataset, consistency values from Granger causality estimates were relatively low, averaging 37.4 +/- 8.6 s.d.

For HCP data, greater than 99% of resting time series passed both KPSS and Dickey-Fuller tests, and only data passing all stationarity testing was included in the analysis. For task-based data, where many fewer volumes were available, between 30% and 70% of the data passed both KPSS and Dickey-Fuller tests, and thus differenced time series were used instead for all tasks. In all 7 tasks, greater than 99% of the differenced time series passed both KPSS and Dickey-Fuller tests for stationarity, and only data that passed both tests was used in analysis.

### Replication in Single Subject Dataset

To assess for the possibility that the results obtained were a consequence of a large heterogenous dataset, in which the only factor consistently observed in Granger causality metrics was vascular confounds, we also analyzed a replication dataset previously obtained that consisted of 100 five-minute fMRI scans in a single subject (male, 39 years old). These were obtained as ten five-minute BOLD sequences obtained in ten separate sessions over a 3-week interval. Half of the sessions were obtained in the resting state with eyes open, and half (5 sessions) were obtained during passive viewing of a cartoon stimulus (Bugs Bunny, Looney Tunes Golden Collection, volVol. 1, Warner Bros.) Scan details have been previously described (TR = 2.0 s, TE = 28 ms, 155 volumes per scan) [19]. The difference time series for each of the 10 sessions was analyzed with KPSS and ADF tests for stationary and only stationary time series were used in the analysis. Granger causality estimates were computed for each of the 50 resting state and 50 cartoon viewing epochs that passed stationarity tests and analogous Granger causality metrics were obtained to those in the larger, more heterogenous dataset, using difference time series for improved stationarity.

## Results

### Reproducibility of Granger Causality

For each of 1240 subjects, Granger causality (GC) was estimated between each pair of 7266 target and 264 seed gray matter ROIs. This resulted in 1,918,224 region pairs. For each of these region pairs, a nonparametric sign test was computed for the difference of target ROI Granger causes seed ROI and seed ROI Granger causes target ROI across 1240 subjects and across each subset of 620 subjects in the test and replication samples. To assess the reproducibility of GC measurements, Z-statistics were compared in the two subject samples (n=620 subjects each). A positive Z-statistic implies that the seed ROI is Granger caused by the target ROI greater than the opposite direction, and a negative Z-statistic implies that the target ROI Granger causes the seed ROI to a greater extent.

**Figure 1:**
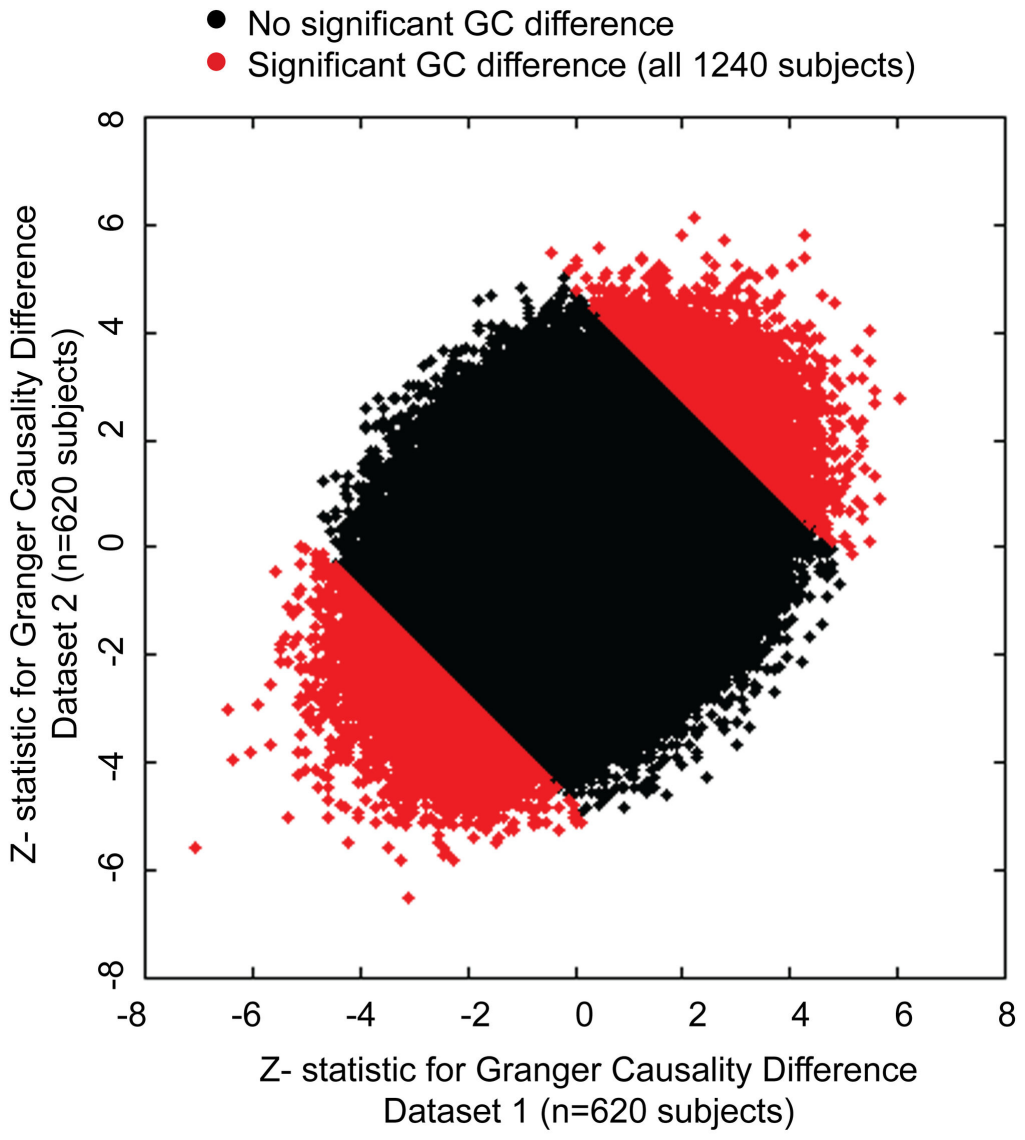
Reproducibility of Granger causality measurements in two subject subsamples. Difference in Granger causality (seed to target minus target to seed) was computed for 7266 target ROI x 264 seed ROI pairs in two subject samples of 620 subjects each. Scatter plot compares Z-statistics for each subject sample across all connections. Dots shown in red exhibited significant Granger causality difference for entire 1240 subject dataset. Dots in blue show connections that were significant in both subject samples after false discovery rate correction for multiple comparisons.

A scatter plot of all the connections tested is shown in Figure **1**. The measurements show reproducibility between the two subject samples, as indicated by clustering of the Z-statistics along the main diagonal. The Spearman correlation coefficient of Z-statistics for the 2 samples was r=0.30, with p-value vanishingly small. In the full sample of 1240 subjects, 1.4% (26602) of possible connections showed significant differences in Granger causality between target and seed ROI with acceptable false discovery rate q<0.05 across all possible connections. Of these, all but 4 connections had the same polarity in both samples. For example, if for a given connection the target ROI Granger caused the seed ROI to a significantly greater extent than the seed ROI Granger caused the target ROI, this was also true in the other sample of subjects. 

**Figure 2:**
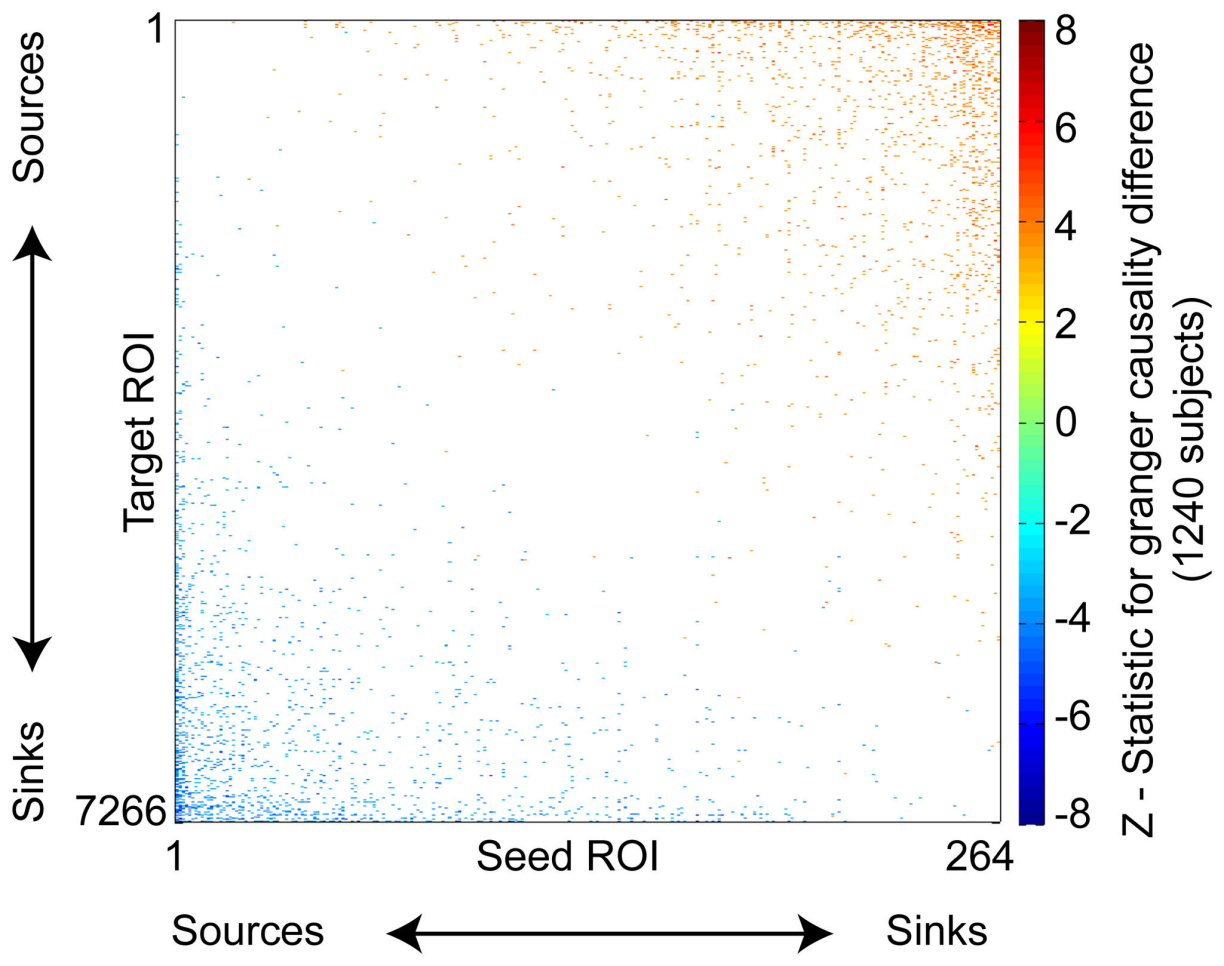
Significant Granger causality differences among seed and target ROIs. Colored squares indicate connections where seed ROI Granger causes target ROI more than target ROI Granger causes seed ROI (blue colored squares) or vice versa (red/orange colored squares), significant across 1240 subjects with false discovery rate correction for multiple comparisons.

Among connections showing significant differences in Granger causality between seed and target ROI, there is a homogeneous progression from Granger sources (which Granger cause other ROIs) to Granger sinks (Granger caused by other ROIs). This is shown by a simple reordering of the seed and target ROIs based on the mean Z-statistic of each ROI with the 264 seeds (including significant connections only), shown in Figure **2**. Essentially all of the significant connections below the main diagonal have negative Z-statistics, and all of the connections above the main diagonal have positive Z-statistics. An ROI is Granger caused by other ROIs with more source-like behavior, and Granger causes ROIs with more sink-like behavior.

### Spatial Distribution of Granger Causality Differences

**Figure 3:**
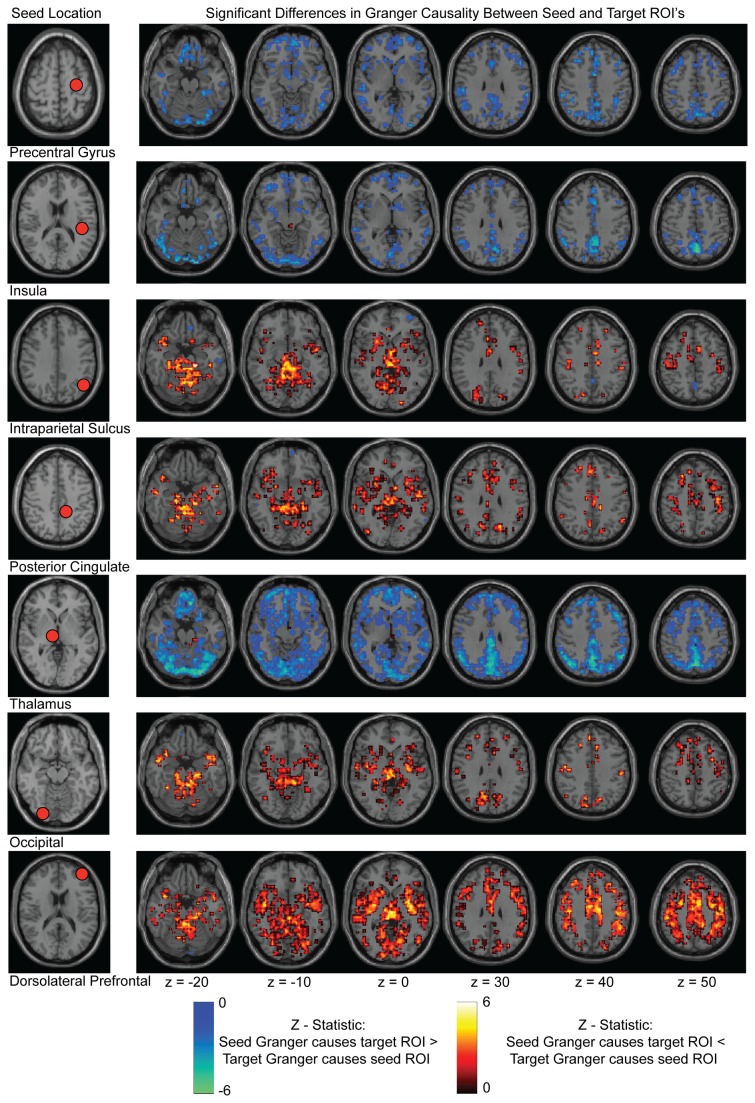
Significant Granger causality differences for 7 seed ROIs to the rest of the gray matter. Seed locations are shown in the leftmost column. Slice locations are reported below the figure in MNI coordinates. Images are in radiologic format with subject left on image right. False discovery rate correction was performed for each seed separately.

The spatial distribution of Granger sources and sinks is informative. Figure **3** demonstrates regions with significant Granger causality relationships to seven seeds selected to represent different functional brain networks. Seeds tend to show either significant relationships to source ROIs (warm colors) or to sink ROIs (cool colors) but not to both, and significant differences tend to be in the same regions for all of the seeds rather than varying by functional network architecture. The seeds included in the figure are: precentral gyrus (sensorimotor network, MNI: x=-21 y=-31 z=61 ), insula (salience or auditory network, MNI: -38 -33 17 ), intraparietal network (dorsal attention network, MNI: -44 -65 35 ), posterior cingulate (default mode network, MNI: -2 -37 44 ), thalamus (MNI: 6 -24 0), occipital (visual network, MNI: 17 -91 -14), and dorsolateral prefrontal cortex (executive network, MNI: -39 51 17). 

**Figure 4:**
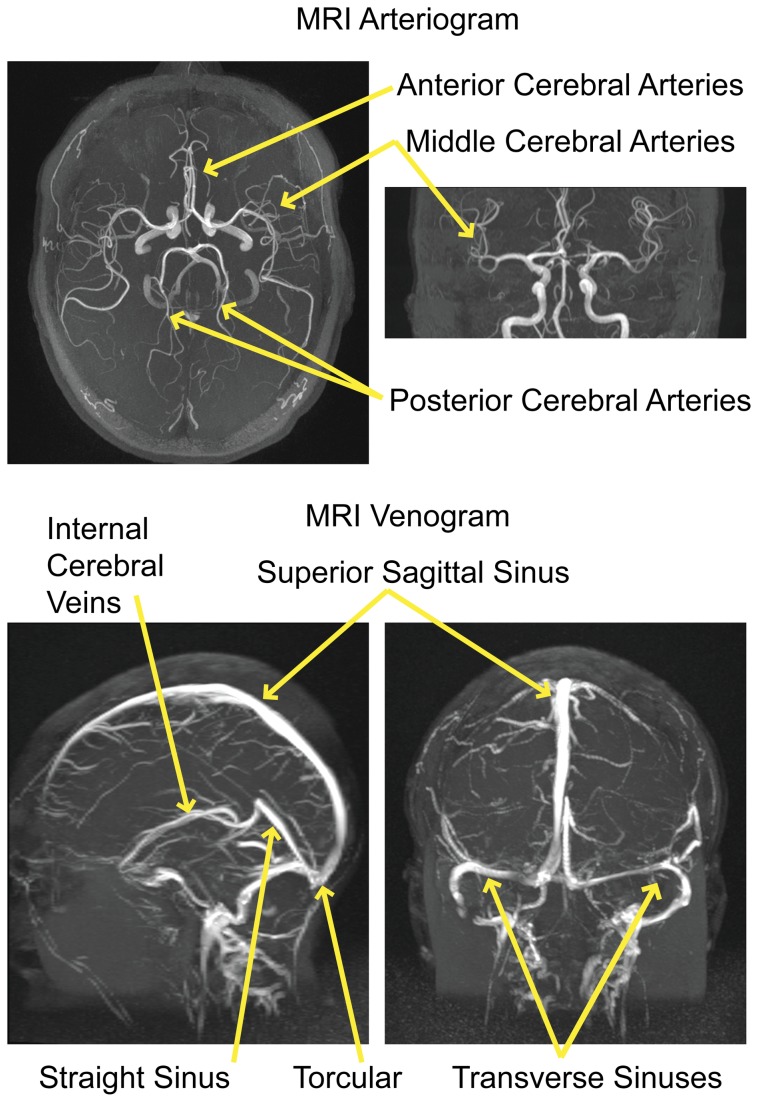
Representative MRI arteriogram (above) and MRI venogram (below) from one subject, with major arteries and venous sinuses labeled, shown in maximum intensity projection for axial and coronal views.

The regions shown in Figure **3** strongly suggest a vascular etiology for Granger causality differences. Granger sources tend to be located centrally within the brain adjacent to the Circle of Willis, and Granger sinks tend to be located peripherally with a spatial distribution that closely matches large venous sinuses such as the transverse and sagittal sinuses. For reference, an MR venogram and MR angiogram for one subject each are shown in Figure **4**, with major arterial inflow distributions and venous outflow distributions labeled. 

**Figure 5:**
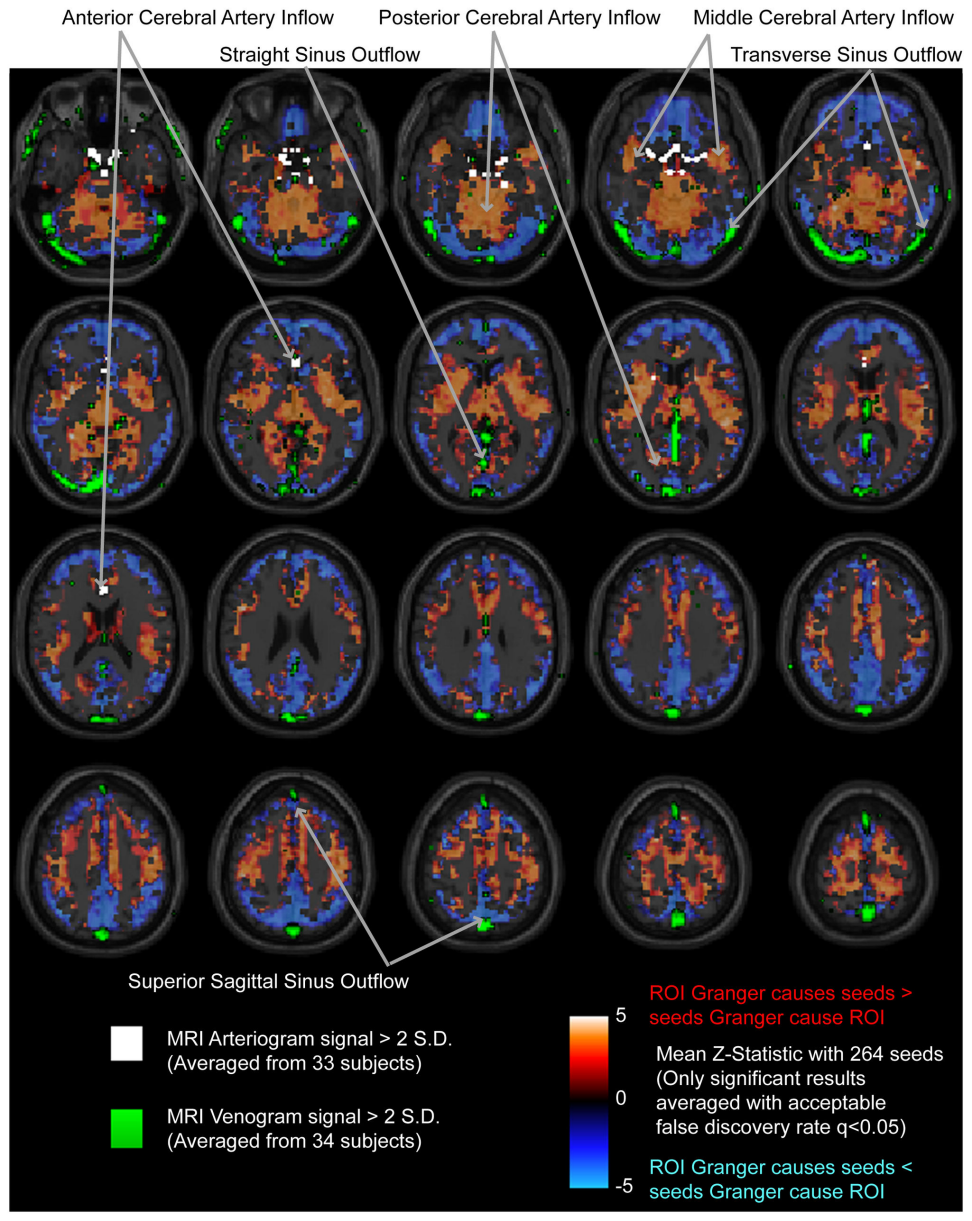
Mean Z-statistic for significant Granger causality differences to seed ROIs. Z-statistics were averaged for a given target ROI with the 264 seed ROIs to which it exhibited significantly asymmetric Granger causality relationship. Masks are overlaid for MRI arteriograms (white) and MRI venograms (green) for voxels with greater than 2 standard deviations signal intensity of in-brain voxels in averaged images from 33 (arteriogram) and 34 (venogram) subjects. Major arterial inflow and venous outflow distributions are labeled.

Large arteries and veins are conserved across individuals in their location, and although there are minor anatomic variations between individuals, the anterior, middle, and posterior cerebral artery territories, and locations of the venous outflow pathways are reproduced from individual to individual. To illustrate the position of these vessels in relationship to Granger sources and sinks noted above, we averaged normalized MRI arteriograms from 33 healthy subjects and MRI venograms from 34 healthy subjects to identify consensus positions of large arteries and veins. We then superimposed the mean Z-statistic of each target ROI with seeds to which it exhibited significant Granger causality differences. The result is shown in Figure **5**. 

Target regions acting as Granger sources (warm colors) reproduce precisely arterial inflow distributions of the anterior, posterior, and middle cerebral arteries, labeled in the figure. Relative Granger sinks (cool colors) in contrast are situated at the periphery of the brain, close to dural venous sinuses. The characteristic spatial distributions of the transverse, straight, and superior sagittal sinuses are faithfully reflected by Granger sink behavior.

Since an ROI appears well-characterized by its ordering from Granger sources to Granger sinks relative to the seeds, dividing the ROIs into deciles allows an animation of the progression of source to sink ROIs, illustrated in the video Figure S1 in Supporting Information. This sequence shows a uniform progression from the Circle of Willis to dural venous sinuses that matches closely what would be expected from blood flowing from arteries to veins in the brain.

**Figure 6:**
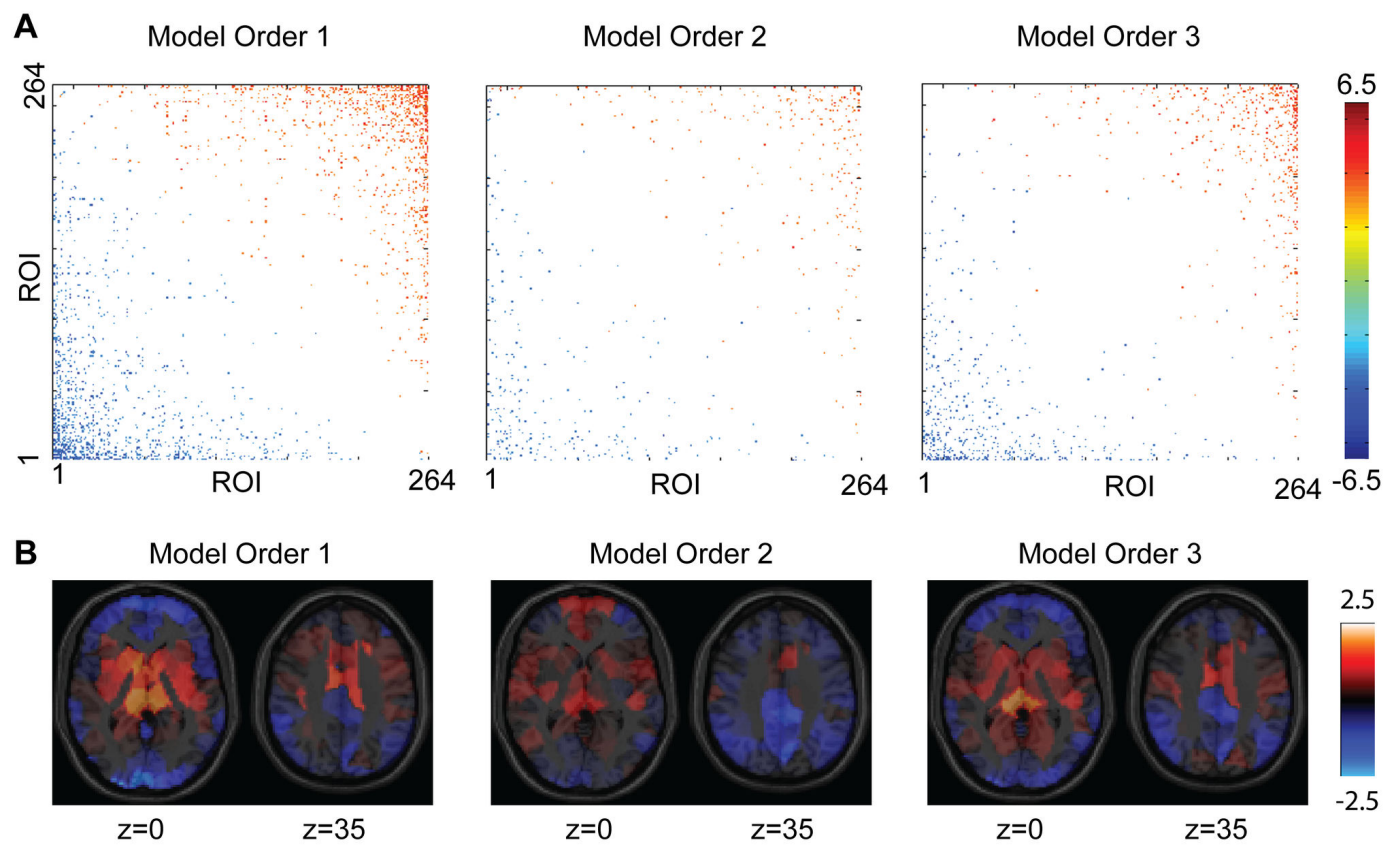
Effect of model order on significant differences in Granger causality. **A**. Significant differences in Granger causality for pairs of 264 seed ROIs were obtained for model orders 1, 2, and 3. Colored squares show pairs of ROIs that significantly differed in forward vs. reverse Granger causality with uncorrected p-value of 0.001 for display. ROIs were ordered from sources to sinks by mean Z-statistic in each case. **B**. Spatial distribution of the mean Z-statistic for Granger causality difference to the other 263 ROIs. Regions shown in red behaved as sources relative to other ROIs and regions shown in blue behaved as sinks relative to other ROIs. Results are shown for model orders 1, 2, and 3. A gray matter mask was colored based on which of the 264 seed ROIs was closest to a given voxel.

Since the dataset studied was obtained from multiple different sites with different acquisition parameters such as repetition time (TR), we evaluated the results for pairs of the 264 seed ROIs at model orders 1, 2, and 3, with results shown in Figure **6**. More significant differences in Granger causality were observed for model orders 1 and 3 than for model order 2, but the results were similar in all 3 cases. Significant differences in Granger causality progressed from source ROIs to sink ROIs in all 3 cases, and the spatial distribution of mean Z-statistic to the 264 seed ROIs showed a similar spatial distribution in all 3 cases with sources located near the Circle of Willis and sinks located near dural venous sinuses and venous outflow territories.

To confirm that the observed results are not a consequence of nonstationarities in the data, we performed a Kwiatkowski-Phillips-Schmidt-Schin test and augmented Dickey-Fuller test for stationarity on each subject’s preprocessed BOLD time series for each of the 264 seed ROIs and for model order 1. For model order 1, 95% of the time series were stationary; 98% were stationary for model order 2; and 98% were stationary for model order 3, by KPSS test. But a Dickey-Fuller test could not exclude a unit root for nearly all of the time series tested.

**Figure 7:**
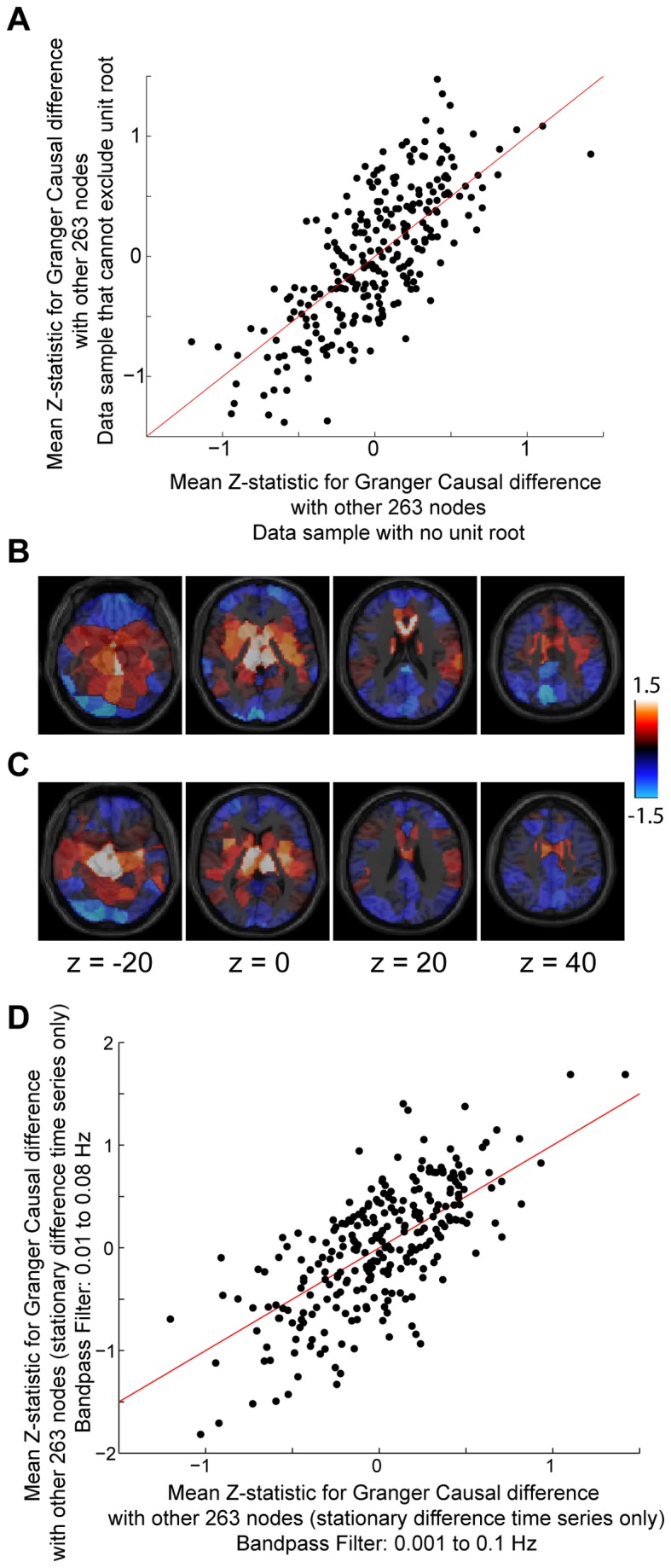
Stationarity of difference time series from preprocessed BOLD data. **A**. Scatterplot shows the mean Z-statistic for 264 seed ROIs with each of the other 263 ROIs for forward – reverse Granger causality. The x-axis values were obtained only from time series satisfying both KPSS and Dickey-Fuller tests of stationarity. The y-axis values were obtained only from time series in which the Dickey-Fuller test could not exclude a unit root. **B**. Mean Z-statistic for each of 264 ROIs with each of the other 263 ROIs for forward – reverse Granger causality, illustrated in 2 axial slices. These data were obtained only from time series that passed stationarity tests. **C**. Similar to above, but obtained from time series that did not pass Dickey-Fuller tests. **D**. For stationary data, comparison of mean Z-statistic for each of the 264 seed ROIs that was obtained using two different bandpass filter widths during preprocessing.

To further evaluate the possibility that nonstationarities may underlie the observed results, we additionally computed the difference time series between each adjacent time point in each of the preprocessed BOLD time series, and repeated all of the Granger causality analyses on the difference time series. For these difference time series, 100% of the data were stationary by KPSS test and 41.6% of the difference time series could exclude a unit root by augmented Dickey-Fuller test. We compared the Granger causality results for difference time series that passed all stationarity tests with those that did not and found equivalent results among the two subsets of data with respect to the spatial distribution of Granger causality differences. These data are shown in Figure **7**, with a scatterplot and axial images showing mean Z-statistic for forward – reverse Granger causality with 264 seed ROIs with the remaining 263 ROIs. In both cases, sources are present in the center of the brain near the Circle of Willis, with sinks present near the dural venous sinuses and in the periphery of the brain. We also tested, for time series passing all stationarity tests only, whether the width of the bandpass filter used in preprocessing could affect the Granger causality results[41] and found that mean Z-statistics (source or sink behavior) was similar for both filter widths (Figure **7D**).

**Figure 8:**
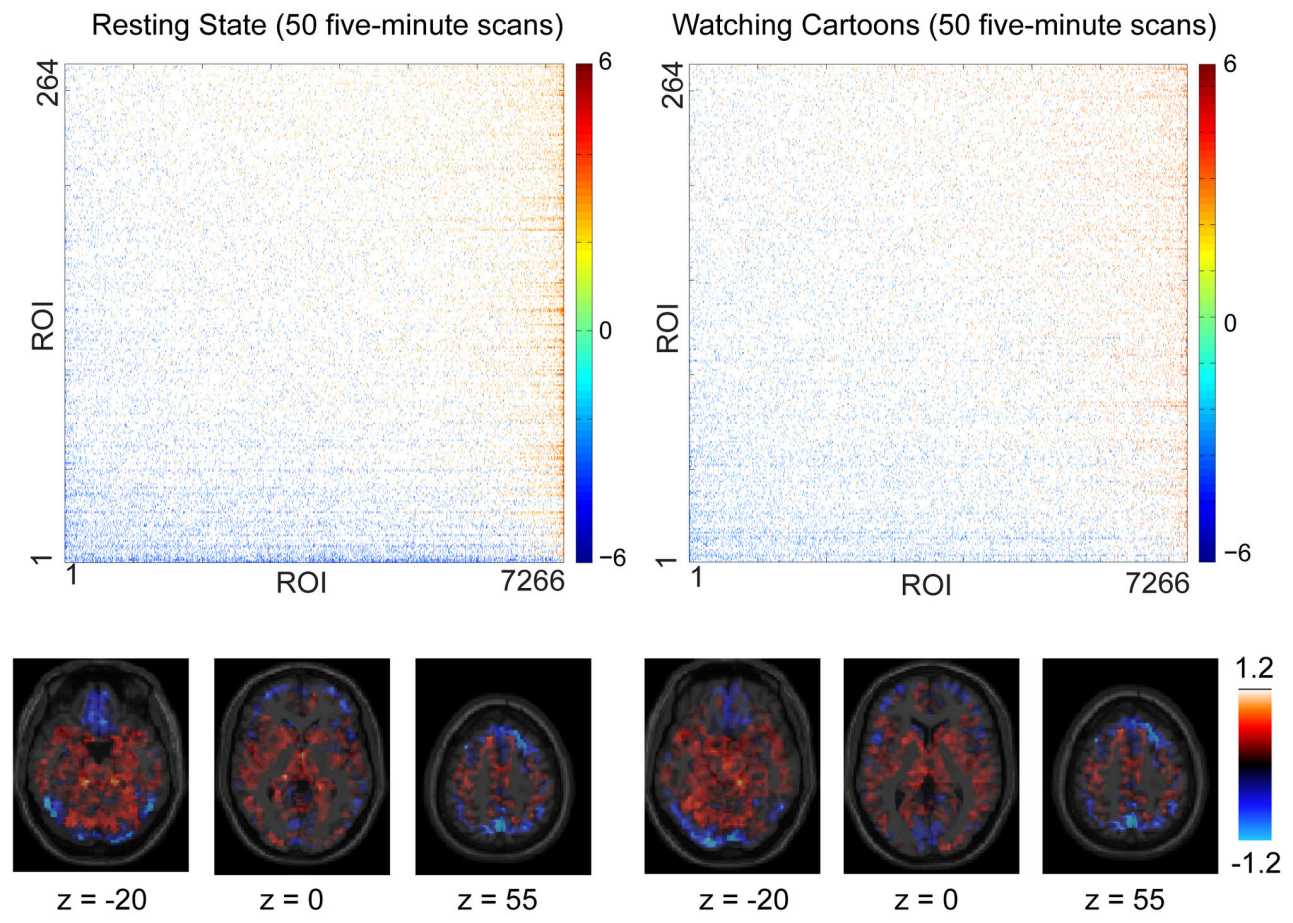
Single subject estimates of Granger causality. Above, Z-statistic for forward – reverse Granger causality for 264 seed ROIs compared to 7266 target ROIs. ROIs were ordered by mean Z-statistic with the remaining ROIs. The left column shows data obtained from 50 5-minute resting state fMRI acquisitions, and the right column shows data obtained from 50 5-minute fMRI acquisitions during passive cartoon viewing. Pseudocolor plot images were thresholded at p<0.001, uncorrected, for display. Below, mean Z-statistic with the 264 seed ROIs for each of 7266 Target ROIs for 3 axial slices with MNI z-coordinate indicated below the images.

As an additional test, we evaluated whether similar results could be obtained from a much more tightly controlled dataset, consisting of 100 5-minute fMRI acquisitions from a single subject over 3 weeks. These data are shown in Figure **8**. Half of the data was obtained with the subject in a resting state and half while the subject passively viewed Bugs Bunny cartoons. For both resting and passive viewing acquisitions, a distribution from source ROIs to sink ROIs was found that was equivalent to that seen in Figure **2** in the larger dataset. The spatial organization of the source ROIs were again seen centrally, with sink ROIs present peripherally, particularly near dural venous sinuses. For the single subject analyses, only difference time series were used that passed both KPSS and Dickey-Fuller stationarity tests (92.1% of the time series).

**Figure 9:**
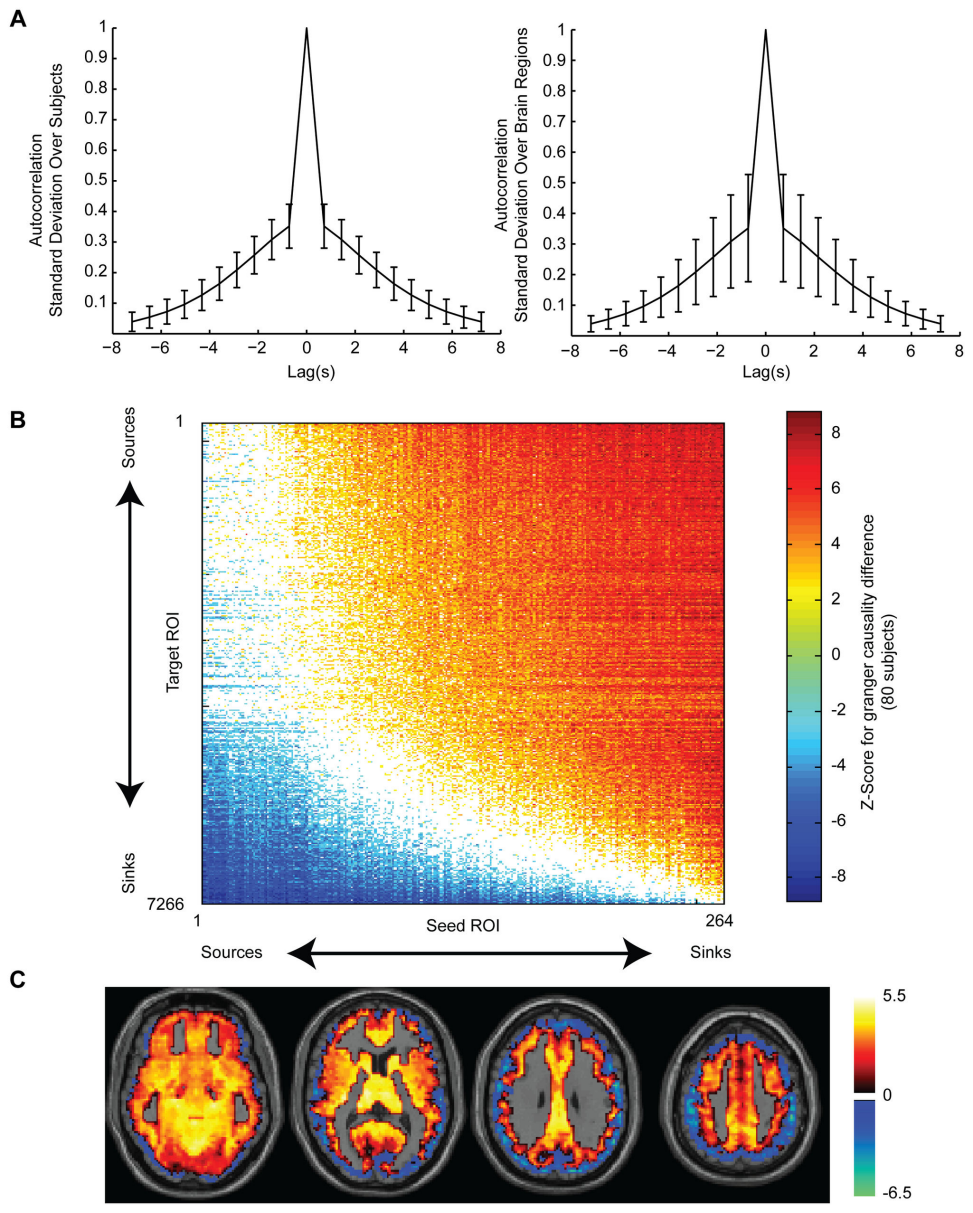
Human Connectome Project multiband resting BOLD Granger causality. **A**. Autocorrelation of time series with error bars indicating standard deviation across subjects (left) and across brain ROIs (right). **B**. Average Z-scores for forward – reverse Granger causality for 80 subjects in 264 seed regions by 7266 target regions matrix. Colored squares show region pairs where a significant difference was obtained with acceptable false discovery rate q<0.05 across all region pairs. **C**. Axial slices at MNI z=-10, 10, 30, and 50 showing mean Z-score of each ROI for forward- reverse Granger causality compared to 264 seed ROIs. Images are in radiological format with subject left on image right.

Given that temporal undersampling may affect Granger causal inferences[22], we tested an independent resting state dataset released by the Human Connectome Project that was acquired using a multiband BOLD sequence with much higher temporal resolution (TR=720 ms). As an additional evaluation of stationarity in this dataset, autocorrelation functions are shown averaged across all time series by subject and by region with standard deviations at each lag reported in Figure **9A**. Autocorrelation returns to baseline over about 8 seconds. For the same 264 x 7266 matrix of ROIs, an equivalent progression from Granger causal sources to Granger causal sinks was observed (Figure **9B**). By evaluating the mean Z-score of each of 7266 ROIs to the 264 seed ROIs for Granger causal difference, images of the brain were obtained with sources in the center of the brain in arterial inflow distributions and sinks at the periphery of the brain where large venous structures are present.

**Figure 10:**
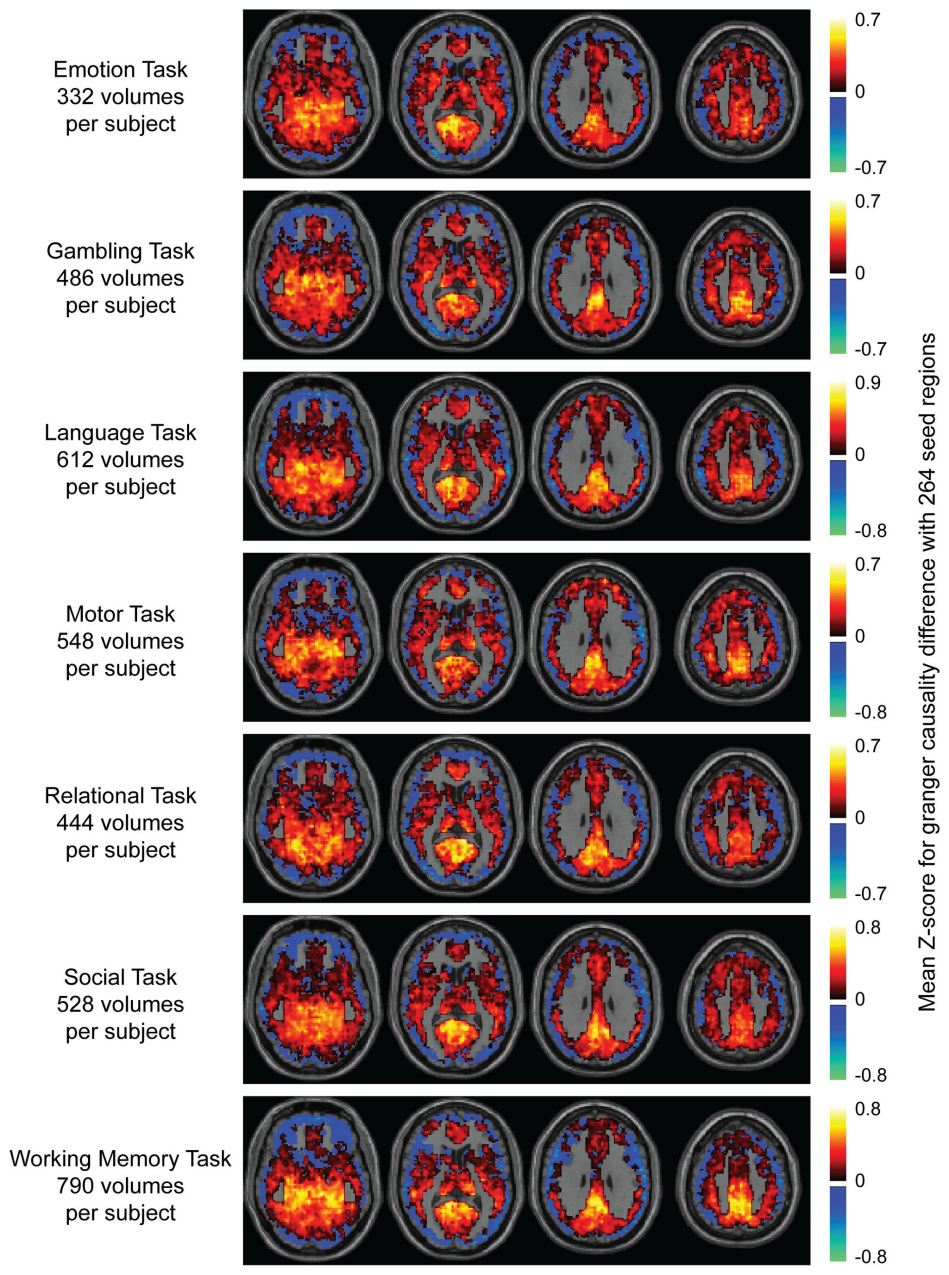
Human Connectome Project task paradigm data. Axial slices at MNI z= -10, 10, 30, and 50 showing mean Z-score of each ROI for forward – reverse Granger causality compared to 264 seed ROIs. The number of volumes used in the analysis for each subject and the task name are shown at the left. Color scale shows mean Z-score for each ROI.

Finally, an analogous procedure was performed on HCP data from the same 80 subjects for each of 7 task paradigm acquisitions, shown in Figure **10**. For all 7 tasks, a progression from sources to sinks was seen with sources in the center of the brain and sinks along the periphery, similar to the resting state data. Although the Z-scores were smaller for task acquisitions due to many fewer volumes used in the analysis than for resting state analysis, the spatial distribution mirrors exactly what was seen in the resting state condition, suggesting the dominant Granger causal effect is explained by the progression from sources to sinks following a vascular flow pattern.

## Discussion

We demonstrate that BOLD Granger causality differences can be reproducibly measured in large samples of resting state fMRI data between brain regions. Gray matter regions can be uniformly ordered from “Granger sources” that Granger cause other ROIs to “Granger sinks” that are Granger caused by other ROIs with more sourcelike behavior. These Granger causal differences show a consistent spatial relationship, with sources near regions associated with arterial inflow, and sinks near regions associated with venous drainage. 

These data can be explained by considering that the BOLD signal is fundamentally a measurement of blood flow. Moreover, the BOLD signal underlying functional connectivity MRI likely represents synchronized amplitude modulation[42] or power fluctuations in neural activity over time[43,44] of brain regions aliased through a hemodynamic filter, resulting in very slow[45] but temporally correlated signals. It is therefore plausible that the pattern of blood flow observed in any one brain region will be recapitulated but temporally delayed at various points along the venous drainage pathway of the brain, since the venous drainage will reflect to some extent the same temporal pattern of blood oxygenation seen in the brain regions drained by the veins. 

Previous reports have noted that systematic variations in end-tidal CO2 associated with depth of respiration can have significant effects on functional connectivity. For example, it has been shown that clamping end-tidal CO2 artificially can improve the specificity of functional connectivity metrics[46]. Attempts to regress out the effects of fluctuations in depth of respiration have similarly shown improvements in accuracy of functional connectivity measures[47,48]. Such fluctuations may be transmitted to the BOLD signal at a variable latency, resulting in artificial sources of lag in the BOLD signal[49]. Indeed, a map of latency obtained using a breath-hold task [49] (Chang et al., 2008, Figure 5) identifies similar brain regions to Granger sinks in Figure 5 of the present report. Our results extend this literature to indicate that such non-neural lag sources may be a dominant feature in Granger causality results.

Such vascular effects are problematic to correct through preprocessing. In our data, the largest lags are for BOLD Granger causality differences for regions close to large veins and dural venous sinuses. Such arterial inflow and venous drainage has a highly reproducible pattern across individuals where major arterial and venous distributions are largely invariant across subjects, giving the illusion of reliable timing differences between brain regions that may be completely unrelated to actual differences in effective connectivity. Significant Granger causality differences in our analysis persisted despite regression of CSF, white matter, and soft tissue time series from each voxel designed to reduce the effects of vascular contamination[28], using a separate independent component analysis correction technique[34], and using only minimal preprocessing[31]. 

Attempts to use measured heart rate and respiratory waveforms as regressors for BOLD data may improve the accuracy of directed functional connectivity measurements, but inherent in these approaches is the problem that the vascular timing differences on the order of seconds that it takes for blood to flow from arteries to sinuses are likely greater in magnitude than neural timing differences for spontaneous brain activity, so correction must be very precise to allow resolution of neural timing differences. Lags between BOLD time series for regions with suspected underlying neural connectivity tend to be close to zero[50]. Even more problematic, measured heart rate and respiratory waveforms are recapitulated throughout the brain at different lags and these lags must be precisely known in order to remove vascular confounds. The use of breath-hold techniques to estimate lags may provide one possible avenue for correction of physiologic artifacts at varying lags[49]. Large-sample resting fMRI data with relevant physiologic waveforms are not currently available to test this possibility.

We also note that this effect is distinct from the previously described heterogeneity of the hemodynamic response function(HRF) in the brain[51]. Problems such as variability of the hemodynamic response function, temporal undersampling of fMRI data, and sparsely acquired data can likely be overcome by Granger causality techniques given sufficient data[22,52,53]. But even if an HRF were perfectly estimated at each voxel in the brain, the mechanism implied in our data is that similarly oxygenated blood arrives at variable time points in the brain independently of any neural activation and will affect lag-based directed functional connectivity measurements. Moreover, blood from one region may then propagate to other regions along the venous drainage pathways also independent of neural to vascular transduction. It is possible that the consistent asymmetries in Granger causality measured in our data may be related to differences in HRF latency in different brain regions, but we consider this less likely given the simpler explanation of blood moving from arteries to veins given the spatial distribution of our results.

Attempts to use lag-based differences in the BOLD signal have been attempted as a solution to establishing directed functional connectivity relationships in the brain. There has been great recent interest in Granger causality methods in particular, which make use of lag-based information, with over 300 citations per year making use of the techniques[14]. Yet the use of Granger causality and vector autoregression has been highlighted as controversial given the difference in timing of neural activity compared to the slow acquisition rates of fMRI[13]. Nevertheless, In a study measuring theoretical limits of temporal accuracy of the BOLD signal, electrical noise was added to measured BOLD data (TR =1.2 s, 1.5 T scanner), with results indicating that as signal to noise ratio (SNR) increased, temporal uncertainty approached 50 ms[54]. Temporal undersampling of the data may present an additional challenge to Granger causality analysis in fMRI data[22]. The application of Granger causality techniques to resting fMRI data has been in increasingly common application in the literature[55-58], with rapidly developing methodological and application studies.

Simulations using lag-based methods have proven ineffective in contrived data at establishing correct directed functional connectivity relationships[20]. Additional work, however has shown that the use of group analyses may alleviate some of these methodological concerns by exploiting small statistical relationships in BOLD phase, allowing detection of neural phase differences as small as 100 ms[21]. A subsequent comment noted that although technically feasible, differences in vascular delays between brain regions may confound efforts to establish directed functional connectivity relationships[59]. Our data demonstrate precisely this scenario, that the dominant feature seen in maps of directed functional connectivity in the resting state may be vascular anatomy rather than sequential neural activation, which may not be simply corrected by regression of vascular time courses, larger sample sizes, or region-specific hemodynamic response modeling.

We emphasize that while the use of large-sample datasets can be a powerful tool for elucidating small statistical relationships, it also carries the possibility of amplifying sources of bias. Multisite data such as those studied in this report include many sources of variability. TR, number of volumes, experimental conditions, slicing, head motion, magnetic field strength, and subject population are all variable across sites. This heterogeneity is both advantageous and problematic because some variables may “average out” due to heterogeneous acquisitions, while others may be reinforced. For example, we cannot exclude the possibility that a more controlled dataset may find consistent differences in resting state Granger causality primarily attributable to sequential neural activation, whereas only the vascular effects were robust enough to be seen in our analysis. We have tested this possibility by additionally examining data from repeated scans from a single subject, with essentially equivalent results. 

Furthermore, it has been demonstrated that temporal correlations between brain regions are variable over time, as demonstrated by sliding window correlation methods[60]. There may be robust Granger causal asymmetries that are temporally unstable, and may average out during extended acquisitions or large datasets. Indeed we find that much of typical resting fMRI data cannot pass the most rigorous tests of stationarity. This pitfall can be overcome to some extent by considering Granger causality of the first order difference of BOLD time series, with essentially equivalent results to the original time series. Further work will be needed to quantify the magnitude of the vascular effect we describe in relation to other acquisition strategies, including resting and additional task paradigms. In data from a single subject we show that the vascular-derived artifacts we observed were present in both resting and task-related data, although the task data was from passive cartoon viewing and the effects may differ in a task where a simpler paradigm is repeated consistently. Yet in acquisitions from 7 different task conditions, the effect was invariant, with strong reproducible spatial distribution of Granger causal inferences.

It should be noted that much of the data used in this analysis was acquired in an undirected resting state. It is possible that during particular cognitive tasks, there may be more consistent phase delays between brain regions than in the resting state. For example, in a task paradigm where one brain region tends to be activated several seconds after another, Granger causality may more accurately reflect the temporal dynamics of neural activity while it does not in undirected resting. Other task based implementations of Granger causality emphasize differences in lag between two experimental states[11,61] that may not be as susceptible to the vascular artifacts highlighted in this report. Still other reports have made conclusions about sequential network activation using Granger causality in an explicit task where temporal differences in activation may be larger than vascular confounds[62]. Nevertheless, these changes may be superimposed on vascular effects such as described in this report and require explicit activation of a brain region, with results only applicable to the particular regions activated by the task. Such an approach may be difficult to apply towards generating a directed functional connectome of the brain. Moreover, in all 8 task conditions we tested, similar results were obtained to the resting state analyses. Further work will be required to determine the extent to which explicit tasks may alleviate the artifacts observed in this report.

## Conclusions

We demonstrate using large, publicly-available resting state and test-retest single subject BOLD datasets that reliable Granger causality differences in BOLD signal can be measured between any two brain regions. Nevertheless, these relationships primarily reflect phase differences attributable to vascular drainage pathways. Granger causality analyses using resting state fMRI to evaluate effective connectivity between BOLD time series in different brain regions should consider the possible effects of systematic differences in arteriovenous anatomy and drainage.

## Supporting Information

Figure S1 **Granger causality sources and sinks.** Target ROIs were divided into deciles based on mean Z-statistic with the 264 seeds and shown in animation from Granger causal sources to Granger causal sinks.
(MP4)
